# Selbstwahrgenommene Mundgesundheit und assoziierte Faktoren bei Erwachsenen in Deutschland. Ergebnisse aus GEDA 2019/2020-EHIS

**DOI:** 10.1007/s00103-021-03376-z

**Published:** 2021-07-07

**Authors:** Laura Krause, Stefanie Seeling, Anne Starker

**Affiliations:** 1 Abteilung für Epidemiologie und Gesundheitsmonitoring, FG 24 Gesundheitsberichterstattung, Robert Koch-Institut, General-Pape-Str. 62–66, 12101 Berlin, Deutschland; 2 Abteilung für Epidemiologie und Gesundheitsmonitoring, FG 27 Gesundheitsverhalten, Robert Koch-Institut, Berlin, Deutschland

**Keywords:** Mundgesundheit, Selbstwahrnehmung, Risikofaktoren, Erwachsene, GEDA, Oral health, Self-perception, Risk factors, Adults, GEDA

## Abstract

**Hintergrund und Ziel der Arbeit:**

Informationen zur Mundgesundheit der Bevölkerung sind wichtig für die Einschätzung von (vermeidbarer) Krankheitslast, für die Einschätzung und Planung von Gesundheitsressourcen und -kosten sowie für die Beurteilung gesundheitlicher Ungleichheiten. Ziel dieser Arbeit ist es, erstmals auf Datenbasis einer bundesweit repräsentativen Stichprobe für die erwachsene Bevölkerung in Deutschland die selbstwahrgenommene Mundgesundheit einschließlich assoziierter Faktoren zu untersuchen.

**Material und Methoden:**

Datenbasis ist die Studie Gesundheit in Deutschland aktuell (GEDA 2019/2020-EHIS, *n* = 22.708 ab 18 Jahre). Im telefonischen Interview wurden die Teilnehmenden gefragt, wie sie den Zustand ihrer Zähne und ihres Zahnfleischs beschreiben würden – „sehr gut“, „gut“, „mittelmäßig“, „schlecht“, „sehr schlecht“. Soziodemografische, verhaltensbezogene und zahnmedizinische Merkmale werden als assoziierte Faktoren untersucht. Ausgewiesen werden Prävalenzen und Ergebnisse multivariater binär-logistischer Regressionen (Odds Ratios, OR).

**Ergebnisse und Diskussion:**

71,4 % der Befragten schätzten ihre Mundgesundheit als sehr gut oder gut ein, 28,6 % als mittelmäßig bis sehr schlecht. Die wichtigsten assoziierten Faktoren waren Beeinträchtigungen beim Kauen und Beißen (OR 4,0), ein unerfüllter zahnmedizinischer Versorgungsbedarf (OR 2,3), männliches Geschlecht (OR 1,5) und ein nicht täglicher Obst- und Gemüsekonsum (OR 1,2), bei Männern zudem eine niedrige Bildung (OR 2,1), das tägliche Rauchen (OR 1,6) und eine nicht jährliche zahnmedizinische Inanspruchnahme (OR 1,4). Aus den Ergebnissen lassen sich Ansatzpunkte zur Förderung der Mundgesundheit ableiten.

## Abstract (en)

**Background and aim of the work:**

Information on the oral health of the population is important for the assessment of (preventable) disease burden, for the estimation and planning of health resources and costs, and for the evaluation of health inequalities. The aim of this work is to investigate for the first time self-perceived oral health, including associated factors, based on data from a nationally representative sample for the adult population in Germany.

**Material and methods:**

The data basis is the nationwide German Health Update study (GEDA 2019/2020-EHIS, *n* = 22,708 aged 18 years and older). In the telephone interview, the participants were asked how they would describe the state of their teeth and gums – “very good,” “good,” “fair,” “bad,” or “very bad.” Sociodemographic, behavioral, and dental characteristics are examined as associated factors. Prevalences and results of multivariate binary logistic regressions (odds ratios, OR) are reported.

**Results and discussion:**

Of the participants, 71.4% perceived their oral health as very good or good, 28.6% as fair to very bad. Difficulty in chewing and biting on hard foods (OR 4.0), unmet dental care needs (OR 2.3), male gender (OR 1.5), and not consuming fruits and vegetables daily (OR 1.2) were the most important associated factors for fair to very bad self-perceived oral health; for men, low education (OR 2,1), daily smoking (OR 1.6) and not receiving dental care annually (OR 1.4) were also important. From the results, starting points for promoting oral health can be derived.

## Hintergrund

Mundgesundheit ist ein zentraler Bestandteil der allgemeinen Gesundheit und von großer Bedeutung für Wohlbefinden und Lebensqualität [1]. Die selbstwahrgenommene Mundgesundheit spiegelt die individuelle Sichtweise der Befragten wider, wobei in die Bewertung neben subjektiven Kriterien (z. B. ästhetische Aspekte, Schmerzen) auch objektive Kriterien (z. B. Munderkrankungen, veränderte Funktionsfähigkeit) eingehen [2]. Die selbstwahrgenommene Mundgesundheit kann in Studien mit einer einfachen Frage erhoben werden [3]. Die europäische Gesundheitsumfrage (European Health Interview Survey – EHIS) hat in der 3. Erhebungswelle das Thema Mundgesundheit aufgegriffen und die Frage zur selbstwahrgenommenen Mundgesundheit in das Modul zum Gesundheitszustand integriert. Als Begründung wird die gesundheitspolitische Relevanz des Themas angeführt: Informationen über den Mundgesundheitsstatus der Bevölkerung sind wichtig für die Einschätzung der (vermeidbaren) Krankheitslast, für die Einschätzung und Planung von Gesundheitsressourcen und -kosten sowie für die Beurteilung möglicher gesundheitlicher Ungleichheiten [4]. Darüber hinaus kann die Analyse der selbstwahrgenommenen Mundgesundheit auf Bevölkerungsebene Ansatzpunkte für Interventionen zur Verbesserung der Mundgesundheit aufzeigen [5]. Wichtig ist es in diesem Zusammenhang, Faktoren zu identifizieren, die mit der selbstwahrgenommenen Mundgesundheit assoziiert sind [6].

International wurde die selbstwahrgenommene Mundgesundheit einschließlich assoziierter Faktoren bereits untersucht. Als Risikofaktoren für eine schlechte selbstwahrgenommene Mundgesundheit im Erwachsenenalter konnten ermittelt werden: männliches Geschlecht [7–9], höheres Alter [7–14], niedriger sozioökonomischer Status bzw. niedrige Bildung, niedriges Einkommen und geringe berufliche Qualifikation [7, 9–11, 14, 15], unzureichende Mundhygiene [8, 9, 13–15], ungesunde Ernährung [10, 13, 15–17], Rauchen [14, 16, 18], Befunde in der Mundhöhle (z. B. Zahnfleischbluten, Zahnverlust, eingeschränkte Kaufunktion) [10–15, 18], geringe zahnärztliche Inanspruchnahme [11, 12, 14, 15, 18–20] sowie ein unerfüllter zahnmedizinischer Versorgungsbedarf [8, 10, 11].

Ziel der vorliegenden Arbeit ist es, erstmals auf Datengrundlage einer bundesweit repräsentativen Stichprobe für die erwachsene Allgemeinbevölkerung in Deutschland die selbstwahrgenommene Mundgesundheit einschließlich assoziierter Faktoren abzubilden und anhand internationaler Befunde einzuordnen. Die differenzierte Auswertung nach soziodemografischen, verhaltensbezogenen und zahnmedizinischen Aspekten erlaubt, Ansatzpunkte zur Förderung der Mundgesundheit in Deutschland aufzuzeigen.

## Methoden

### Studiendesign

Datengrundlage ist die Studie Gesundheit in Deutschland aktuell (GEDA). GEDA ist eine bundesweite Befragung der in Deutschland lebenden Wohnbevölkerung, die vom Robert Koch-Institut (RKI) in mehrjährigen Abständen durchgeführt wird [21]. Sie ist Teil des bevölkerungsbezogenen Gesundheitsmonitorings am RKI, dessen Aufgabe es ist, zuverlässige Informationen über die gesundheitliche Lage der Bevölkerung bereitzustellen. Seit 2008 wurden 5 GEDA-Wellen realisiert. Datenbasis der vorliegenden Analyse ist die aktuelle Welle GEDA 2019/2020-EHIS, die zwischen April 2019 und September 2020 als telefonische Befragung stattgefunden hat. Insgesamt wurden 23.001 Personen ab 15 Jahren interviewt, die entweder über Festnetz oder Mobilfunk erreichbar waren (Response: 22,0 %). Seit der 4. Erhebungswelle wird in GEDA der Fragebogen des EHIS eingebettet [22, 23]. In GEDA 2019/2020-EHIS wurde der Fragebogen der 3. EHIS-Welle vollständig integriert, dessen Fragen und Formulierungen verpflichtend sind [4]. Dieser besteht aus 4 Modulen zu folgenden Aspekten: Gesundheitszustand, Gesundheitsversorgung, Gesundheitsdeterminanten sowie demografische und sozioökonomische Charakteristika der Teilnehmenden. Eine ausführliche Beschreibung der EHIS-Studie ist an anderer Stelle publiziert [22, 23].

### Zielvariable

#### Selbstwahrgenommene Mundgesundheit.

Die Frage zur selbstwahrgenommenen Mundgesundheit aus dem EHIS lautet: „Nun folgt eine Frage zum Thema Mundgesundheit. Wie würden Sie den Zustand Ihrer Zähne und Ihres Zahnfleischs beschreiben? – ‚sehr gut‘, ‚gut‘, ‚mittelmäßig‘, ‚schlecht‘, ‚sehr schlecht‘.“ Für die Analysen wurde eine Dichotomisierung der Antwortkategorien in „sehr gut/gut“ vs. „mittelmäßig/schlecht/sehr schlecht“ vorgenommen. Um die selbstwahrgenommene Mundgesundheit differenzierter beleuchten zu können, wurden neben dem Geschlecht der Befragten weitere Merkmale zur Stratifizierung herangezogen. Diese werden im Folgenden einschließlich Fragestellung, Antwortoptionen und, im Einklang mit früheren RKI-Publikationen, vorgenommener Dichotomisierung dargestellt:

### Soziodemografische Merkmale

#### Alter und Bildung.

Für die Auswertungen erfolgte die Einteilung in Altersgruppen unter Berücksichtigung der Empfehlung der Weltgesundheitsorganisation (WHO; 35–44 Jahre, 65–74 Jahre) [24]. Die Internationale Standardklassifikation für das Bildungswesen (ISCED) wurde verwendet, um die schulischen und beruflichen Bildungsabschlüsse der Befragten zu klassifizieren [25].

### Verhaltensbezogene Merkmale

#### Obst- und Gemüsekonsum.

Die Teilnehmenden wurden gefragt: „Wie oft essen Sie Obst? Mit einzubeziehen ist ebenfalls getrocknetes, Tiefkühl- und Dosenobst. Nicht gemeint sind hier Obstsäfte.“ und „Wie oft essen Sie Gemüse oder Salat? Mit einzubeziehen ist getrocknetes, Tiefkühl- und Dosengemüse. Zählen Sie Kartoffeln und Gemüsesäfte bitte nicht mit.“ Die Antwortmöglichkeiten waren jeweils: „täglich oder mehrmals täglich“, „4- bis 6‑mal pro Woche“, „1- bis 3‑mal pro Woche“, „weniger als 1‑mal pro Woche“, „nie“. Um einen täglichen Obst- und Gemüsekonsum abbilden zu können, wurden die Angaben zusammengefasst und die 5‑stufige Antwortskala dichotomisiert in „täglich“ vs. „nicht täglich“ [26, 27].

#### Verzehr zuckerhaltiger Getränke.

Den Teilnehmenden wurde die Frage gestellt: „Wie oft trinken Sie zuckerhaltige Getränke wie gesüßte Fruchtsaftgetränke, Limonade, Cola oder andere zuckerhaltige Erfrischungsgetränke? Bitte zählen Sie Light- und Diätgetränke oder Getränke mit Süßstoff nicht mit.“ Analog dem Obst- und Gemüsekonsum wurde die 5‑stufige Antwortskala dichotomisiert in „täglich“ vs. „nicht täglich“.

#### Tabakkonsum.

Die Teilnehmenden wurden gefragt: „Rauchen Sie Tabakprodukte, einschließlich Tabakerhitzer? Bitte schließen Sie elektronische Zigaretten oder ähnliche Produkte aus. Mit Tabakerhitzern sind z. B. IQOS oder HEETS Tabaksticks gemeint.“ Die Antwortmöglichkeiten lauteten: „ja, täglich“, „ja, gelegentlich“, „nein, nicht mehr“, „Ich habe noch nie geraucht“. Für die Analysen wurden die letzten beiden Kategorien zusammengefasst. Auf diese Weise wird zwischen täglichem und gelegentlichem Tabakkonsum sowie Personen, die nicht rauchen, unterschieden [28].

### Zahnmedizinische Aspekte

#### Beeinträchtigungen beim Kauen und Beißen.

Die Teilnehmenden ab 55 Jahren wurden gefragt: „Haben Sie Schwierigkeiten mit dem Kauen oder Beißen fester Nahrungsmittel, z. B. von einem Apfel? Würden Sie sagen – ‚keine Schwierigkeiten‘, ‚einige Schwierigkeiten‘, ‚große Schwierigkeiten‘, ‚Es ist mir nicht möglich‘.“ Für die Analysen wurden die letzten 3 Kategorien zusammengefasst. Dadurch erhält man den Indikator Beeinträchtigungen beim Kauen und Beißen (Ja/Nein) [29].

#### Zahnmedizinische Inanspruchnahme.

Den Teilnehmenden wurde die Frage gestellt: „Wann waren Sie zuletzt bei einem Zahnarzt, Kieferorthopäden oder einem anderen zahnmedizinischen Spezialisten, um sich selbst beraten, untersuchen oder behandeln zu lassen?“ Die Antwortmöglichkeiten waren: „vor weniger als 6 Monaten“, „vor 6 bis weniger als 12 Monaten“, „vor 12 Monaten oder länger“, „nie“. Für die Analysen wurden die ersten und die letzten beiden Antwortkategorien zusammengefasst. Auf diese Weise erhält man den Indikator 12-Monats-Prävalenz der zahnmedizinischen Inanspruchnahme (Ja/Nein) [30].

#### Unerfüllter zahnmedizinischer Versorgungsbedarf.

Die Teilnehmenden wurden gefragt: „Kam es in den letzten 12 Monaten vor, dass Sie eine der folgenden Untersuchungen oder Behandlungen benötigt hätten, Sie sich diese aber nicht leisten konnten?“ Unter mehreren Antwortmöglichkeiten konnte „zahnärztliche oder kieferorthopädische Untersuchung oder Behandlung“ angekreuzt werden. Die Antwortkategorien waren: „ja“, „nein“, „kein Bedarf“. Personen, die angaben, in den letzten 12 Monaten keinen Bedarf gehabt zu haben, wurden von den Analysen ausgeschlossen [31].

### Statistische Analyse

Die Auswertungen basieren auf Daten von 22.708 Teilnehmenden ab 18 Jahren mit gültigen Angaben zur selbstwahrgenommenen Mundgesundheit. Tab. 1 zeigt die Verteilung der Stichprobe anhand zentraler Analysemerkmale.

**Table 1:** 

	Fallzahl (*n*)	Ungewichtete Stichprobe (%)	Gewichtete Stichprobe (%)^a^
*Geschlecht*			
Frauen	10.740	47,3	48,9
Männer	11.968	52,7	51,1
*Altersgruppe*			
18–34 Jahre	3011	13,3	22,9
35–44 Jahre	2617	11,5	14,8
45–64 Jahre	8723	38,4	34,9
65–74 Jahre	4573	20,1	13,1
75 Jahre und älter	3784	16,7	14,3
*Bildungsgruppe*			
Untere	1339	5,9	17,8
Mittlere	9669	42,6	57,1
Obere	11.637	51,3	25,1
Fehlende Werte	63	0,3	–
*Selbstwahrgenommene Mundgesundheit*			
Sehr gut	4452	19,9	18,0
Gut	12.309	54,0	53,3
Mittelmäßig	4871	21,3	22,6
Schlecht	828	3,7	4,9
Sehr schlecht	187	0,8	1,1
Fehlende Werte	61	0,3	–

^a^Hochgerechnet auf die erwachsene Wohnbevölkerung Deutschlands zum Zeitpunkt 31.12.2018

Die Daten wurden deskriptiv und mittels logistischer Regression analysiert, wobei die dichotomisierte selbstwahrgenommene Mundgesundheit die abhängige Zielvariable war. Ausgewiesen wurden Prävalenzen mit 95 %-Konfidenzintervallen (95 %-KI) stratifiziert nach soziodemografischen, verhaltensbezogenen und zahnmedizinischen Merkmalen. Mithilfe multivariater binär-logistischer Regressionen wurden zunächst für jedes einzelne Merkmal mit Adjustierung für Geschlecht, Alter und Bildung Odds Ratios (OR) als Effektschätzer für eine mittelmäßige bis sehr schlechte selbstwahrgenommene Mundgesundheit mit 95 %-KI berechnet. Anschließend wurde eine multivariate Regressionsanalyse über alle Stratifizierungsmerkmale durchgeführt (multivariates Gesamtmodell). Ein statistisch signifikanter Unterschied zwischen Gruppen lag vor, wenn der ermittelte *p*-Wert kleiner als 0,05 war. Die Analysen wurden mit einem Gewichtungsfaktor durchgeführt, der Abweichungen der Stichprobe von der Bevölkerungsstruktur (Stand: 31.12.2018) in Bezug auf Geschlecht, Alter, Kreistyp und Bildung (ISCED) korrigiert. Der Kreistyp spiegelt den Grad der Urbanisierung wider und entspricht der regionalen Verteilung in Deutschland. Sämtliche Analysen wurden mit den Surveyprozeduren von Stata 17.0 durchgeführt.

## Ergebnisse

Fast drei Viertel der Erwachsenen (71,4 %; 95 %-KI 70,5–72,3) schätzten ihre Mundgesundheit als sehr gut oder gut ein, etwas mehr als ein Viertel (28,6 %; 95 %-KI 27,7–29,5) als mittelmäßig bis sehr schlecht. Im Vergleich zu Frauen (24,6 %; 95 %-KI 23,5–25,8) berichteten Männer (32,8 %; 95 %-KI 31,4–34,2) häufiger von einer mittelmäßigen bis sehr schlechten Mundgesundheit. Insgesamt war das Risiko, die Mundgesundheit als mittelmäßig bis sehr schlecht einzuschätzen, bei Männern 1,7-mal höher als bei Frauen (*p* < 0,001).

Mit zunehmendem Alter stieg der Anteil derjenigen, die eine mittelmäßige bis sehr schlechte Mundgesundheit berichteten, von 18,5 % bei den 18- bis 34-Jährigen auf 33,9 % bei den 45- bis 64-Jährigen. Im weiteren Altersverlauf stagnierte dieser Anteil auf hohem Niveau. Im Vergleich zu den 18- bis 34-Jährigen hatten 45-Jährige und Ältere ein mehr als 2‑fach erhöhtes Risiko, die Mundgesundheit als mittelmäßig bis sehr schlecht einzuschätzen (*p* < 0,001). Tab. 2 zeigt, dass die Altersunterschiede bei Frauen stärker ausgeprägt waren als bei Männern.

**Table 2:** 

	Frauen			Männer			Gesamt		
	% (95 %-KI)	OR (95 %-KI)	*p*	% (95 %-KI)	OR (95 %-KI)	*p*	% (95 %-KI)	OR (95 %-KI)	*p*
*Altersgruppe*									
18–34 Jahre	13,6 (11,2–16,3)	Ref.	–	23,1 (20,3–26,1)	Ref.	–	18,5 (16,7–20,6)	Ref.	–
35–44 Jahre	19,9 (16,8–23,3)	1,7 (1,2–2,3)	< 0,001	28,3 (24,6–32,3)	1,5 (1,1–1,9)	0,004	24,1 (21,7–26,8)	1,5 (1,3–1,9)	< 0,001
45–64 Jahre	28,8 (26,9–30,8)	2,6 (2,1–3,3)	< 0,001	39,1 (36,7–41,5)	2,4 (2,0–2,9)	< 0,001	33,9 (32,4–35,5)	2,5 (2,1–2,9)	< 0,001
65–74 Jahre	28,0 (25,4–30,8)	2,4 (1,9–3,2)	< 0,001	36,5 (33,3–39,8)	2,2 (1,8–2,8)	< 0,001	32,0 (29,9–34,2)	2,3 (1,9–2,7)	< 0,001
75 Jahre und älter	31,7 (28,5–35,0)	2,7 (2,0–3,5)	< 0,001	35,6 (32,0–39,3)	2,0 (1,6–2,5)	< 0,001	33,3 (30,9–35,8)	2,2 (1,9–2,7)	< 0,001
*Bildungsgruppe*									
Untere	31,9 (28,3–35,7)	2,0 (1,6–2,4)	< 0,001	44,1 (38,9–49,5)	2,9 (2,3–3,7)	< 0,001	36,6 (33,6–39,8)	2,5 (2,1–2,9)	< 0,001
Mittlere	24,5 (23,1–26,0)	1,4 (1,3–1,6)	< 0,001	34,5 (32,5–36,5)	1,7 (1,6–2,0)	< 0,001	29,3 (28,1–30,5)	1,6 (1,5–1,8)	< 0,001
Obere	17,8 (16,6–19,2)	Ref.	–	24,1 (22,8–25,5)	Ref.	–	21,5 (20,5–22,5)	Ref.	–

*OR* Odds Ratios, *95* *%-KI* 95 %-Konfidenzintervall, *Ref.* Referenzgruppe

Hinsichtlich der selbstwahrgenommenen Mundgesundheit zeigt sich ein ausgeprägter Bildungsgradient: Erwachsene der unteren Bildungsgruppe schätzten ihre Mundgesundheit häufiger als mittelmäßig bis sehr schlecht ein als Personen der mittleren Bildungsgruppe, die wiederum häufiger eine mittelmäßige bis sehr schlechte Mundgesundheit berichteten als Erwachsene der oberen Bildungsgruppe. Insgesamt war das Risiko, die Mundgesundheit als mittelmäßig bis sehr schlecht einzuschätzen, bei Personen mit niedriger Bildung um den Faktor 2,5 und bei Personen mit mittlerer Bildung um den Faktor 1,6 erhöht im Vergleich zu Personen mit hoher Bildung (*p* < 0,001). Tab. 2 zeigt, dass die Bildungsunterschiede bei Männern stärker ausfielen als bei Frauen.

Personen, die nicht täglich Obst und Gemüse essen, berichteten häufiger eine mittelmäßige bis sehr schlechte Mundgesundheit als Erwachsene, die das täglich tun (OR 1,4; 95 %-KI 1,3–1,5; *p* < 0,001). Erwachsene, die täglich zuckerhaltige Getränke konsumieren, beurteilten ihre Mundgesundheit häufiger als mittelmäßig bis sehr schlecht als diejenigen mit einem nicht täglichen Konsum. Insgesamt war das Risiko, die Mundgesundheit als mittelmäßig bis sehr schlecht einzuschätzen, bei Personen mit täglichem Konsum zuckerhaltiger Getränke um rund das 2‑Fache erhöht (OR 1,9; 95 %-KI 1,6–2,2; *p* < 0,001). Die Zusammenhänge zwischen der selbstwahrgenommenen Mundgesundheit und dem Ernährungsverhalten waren bei Frauen und Männern etwa gleich stark ausgeprägt (Tab. 3).

**Table 3:** 

	Frauen			Männer			Gesamt		
	% (95 %-KI)	OR (95 %-KI)	*p*	% (95 %-KI)	OR (95 %-KI)	*p*	% (95 %-KI)	OR (95 %-KI)	*p*
*Obst- und Gemüseverzehr*									
Täglich	21,6 (20,1–23,2)	Ref.	–	26,2 (23,8–28,8)	Ref.	–	23,1 (21,8–24,5)	Ref.	–
Nicht täglich	27,1 (25,4–28,9)	1,3 (1,2–1,5)	< 0,001	34,7 (33,1–36,4)	1,5 (1,2–1,7)	< 0,001	31,5 (30,3–32,7)	1,4 (1,3–1,5)	< 0,001
*Konsum zuckerhaltiger Getränke*									
Nicht täglich	23,8 (22,6–25,0)	Ref.	–	30,3 (28,8–31,7)	Ref.	–	26,8 (25,8–27,7)	Ref.	–
Täglich	34,9 (29,6–40,6)	1,8 (1,4–2,4)	< 0,001	45,1 (41,2–49,0)	1,9 (1,6–2,3)	< 0,001	41,7 (38,5–45,0)	1,9 (1,6–2,2)	< 0,001
*Tabakkonsum*									
Nie	23,0 (21,7–24,1)	Ref.	–	27,9 (26,4–29,4)	Ref.	–	25,2 (24,2–26,2)	Ref.	–
Gelegentlich	17,0 (12,3–23,1)	0,9 (0,6–1,3)	0,541	27,9 (22,6–33,9)	1,2 (0,9–1,7)	0,162	23,4 (19,6–27,7)	1,1 (0,8–1,4)	0,540
Täglich	32,7 (29,5–35,9)	1,7 (1,4–2,0)	< 0,001	45,5 (42,3–48,7)	2,2 (1,9–2,6)	< 0,001	40,0 (37,7–42,2)	2,0 (1,8–2,2)	< 0,001

*OR* Odds Ratios, *95* *%-KI* 95 %-Konfidenzintervall, *Ref.* Referenzgruppe

Erwachsene, die täglich rauchen, schätzten ihre Mundgesundheit häufiger als mittelmäßig bis sehr schlecht ein als nicht rauchende Personen. Insgesamt war das Risiko, die Mundgesundheit als mittelmäßig bis sehr schlecht einzuschätzen, bei täglichem Tabakkonsum um das 2‑Fache erhöht (OR 2,0; 95 %-KI 1,8–2,2 *p* < 0,001). Bei Männern fiel dieser Effekt etwas stärker aus als bei Frauen. Für einen gelegentlichen Tabakkonsum zeigte sich kein erhöhtes Risiko für eine mittelmäßige bis sehr schlechte selbstwahrgenommene Mundgesundheit. Dies traf auf beide Geschlechter zu (Tab. 3).

Personen ab 55 Jahren, die Beeinträchtigungen beim Kauen oder Beißen fester Nahrungsmittel haben, gaben mehr als doppelt so häufig eine mittelmäßige bis sehr schlechte Mundgesundheit an wie gleichaltrige Personen ohne solche Schwierigkeiten. Insgesamt war das Risiko, die Mundgesundheit als mittelmäßig bis sehr schlecht einzuschätzen, bei diesen Personen um das 4,5-Fache erhöht (*p* < 0,001).

Erwachsene, die sich in den letzten 12 Monaten nicht in einer zahnärztlichen oder kieferorthopädischen Praxis vorgestellt haben, berichteten häufiger eine mittelmäßige bis sehr schlechte Mundgesundheit als Personen mit entsprechender Inanspruchnahme (OR 1,8; 95 %-KI 1,6–2,0; *p* < 0,001). Personen, die nach eigenen Angaben im letzten Jahr eine zahnärztliche oder kieferorthopädische Untersuchung oder Behandlung benötigt hätten, sich diese aber nicht leisten konnten, gaben mehr als doppelt so häufig eine mittelmäßige bis sehr schlechte Mundgesundheit an als Personen ohne solche Probleme. Insgesamt war das Risiko, die Mundgesundheit als mittelmäßig bis sehr schlecht zu beurteilen, bei diesen Personen um das 3,4-Fache erhöht (*p* < 0,001). Die Zusammenhänge zwischen der selbstwahrgenommenen Mundgesundheit und den zahnmedizinischen Faktoren fielen bei Frauen und Männern etwa gleich stark aus (Tab. 4).

**Table 4:** 

	Frauen			Männer			Gesamt		
	% (95 %-KI)	OR (95 %-KI)	*p*	% (95 %-KI)	OR (95 %-KI)	*p*	% (95 %-KI)	OR (95 %-KI)	*p*
*Beeinträchtigungen beim Kauen und Beißen (ab 55 Jahre)*									
Nein	23,5 (21,9–25,3)	Ref.	–	31,8 (29,8–33,9)	Ref.	–	27,4 (26,1–28,8)	Ref.	–
Ja	58,1 (53,6–62,5)	4,6 (3,8–5,7)	< 0,001	67,2 (61,9–72,1)	4,3 (3,3–5,5)	< 0,001	62,0 (58,6–65,3)	4,5 (3,8–5,3)	< 0,001
*Zahnmedizinische Inanspruchnahme (12-MP)*									
Ja	23,0 (21,8–24,3)	Ref.	–	29,7 (28,2–31,3)	Ref.	–	26,2 (25,2–27,1)	Ref.	–
Nein	34,3 (30,5–38,3)	1,7 (1,4–2,0)	< 0,001	44,3 (40,9–47,7)	1,9 (1,6–2,2)	< 0,001	40,1 (37,6–42,8)	1,8 (1,6–2,0)	< 0,001
*Ungedeckter zahnmedizinischer Versorgungsbedarf (12-MP)*									
Nein	23,4 (22,0–24,8)	Ref.	–	32,0 (30,3–33,6)	Ref.	–	27,5 (26,4–28,6)	Ref.	–
Ja	49,0 (43,6–54,5)	3,4 (2,7–4,3)	< 0,001	60,6 (54,5–66,4)	3,5 (2,6–4,6)	< 0,001	54,4 (50,3–58,4)	3,4 (2,9–4,1)	< 0,001

*OR* Odds Ratios, *95* *%-KI* 95 %-Konfidenzintervall, *Ref.* Referenzgruppe, *12-MP* 12-Monats-Prävalenz

Wurden alle Merkmale gemeinsam in einem Modell berücksichtigt (multivariates Gesamtmodell), zeigt sich, dass Beeinträchtigungen beim Kauen und Beißen (OR 4,0; 95 %-KI 3,4–4,9), ein unerfüllter zahnmedizinischer Versorgungsbedarf (OR 2,3; 95 %-KI 1,8–3,1), männliches Geschlecht (OR 1,5; 95 %-KI 1,3–1,7) und ein nicht täglicher Obst- und Gemüsekonsum (OR 1,2; 95 %-KI 1,1–1,4) die wichtigsten assoziierten Faktoren für eine mittelmäßige bis sehr schlechte selbstwahrgenommene Mundgesundheit waren. Bei Männern war nach statistisch wechselseitiger Kontrolle aller Merkmale zusätzlich niedrige Bildung (OR 2,1; 95 %-KI 1,3–3,3), das tägliche Rauchen (OR 1,6; 95 %-KI 1,1–1,9) und eine nicht jährliche zahnmedizinische Inanspruchnahme (OR 1,4; 95 %-KI 1,6–2,2) mit einer mittelmäßigen bis sehr schlechten selbstwahrgenommenen Mundgesundheit assoziiert (Tab. 5).

**Table 5:** 

	Frauen		Männer		Gesamt	
	OR (95 %-KI)	*p*	OR (95 %-KI)	*p*	OR (95 %-KI)	*p*
*Geschlecht*						
Frauen	–	–	–	–	Ref.	–
Männer	–	–	–	–	1,5 (1,3–1,7)	< 0,001
*Altersgruppe*						
55–64 Jahre	Ref.	–	Ref.	–	Ref.	–
65–74 Jahre	0,8 (0,6–1,0)	0,043	1,0 (0,8–1,2)	0,920	0,9 (0,7–1,0)	0,076
75 Jahre und älter	0,9 (0,7–1,1)	0,193	0,9 (0,7–1,1)	0,317	0,8 (0,7–1,0)	0,057
*Bildungsgruppe*						
Untere	1,1 (0,9–1,5)	0,412	2,1 (1,3–3,3)	0,002	1,4 (1,1–1,8)	0,005
Mittlere	1,0 (0,9–1,2)	0,668	1,1 (1,0–1,3)	0,137	1,1 (1,0–1,3)	0,055
Obere	Ref.	–	Ref.	–	Ref.	–
*Obst- und Gemüseverzehr*						
Täglich	Ref.	–	Ref.	–	Ref.	–
Nicht täglich	1,3 (1,0–1,5)	0,021	1,3 (1,0–1,6)	0,035	1,2 (1,1–1,4)	0,003
*Konsum zuckerhaltiger Getränke*						
Nicht täglich	Ref.	–	Ref.	–	Ref.	–
Täglich	1,3 (0,8–2,0)	0,256	1,0 (0,7–1,4)	0,904	1,1 (0,9–1,4)	0,447
*Tabakkonsum*						
Nie	Ref.	–	Ref.	–	Ref.	–
Gelegentlich	0,6 (0,3–0,9)	0,031	1,4 (0,8–2,4)	0,207	0,9 (0,6–1,4)	0,687
Täglich	1,2 (0,9–1,5)	0,181	1,6 (1,2–2,1)	< 0,001	1,4 (1,1–1,7)	< 0,001
*Beeinträchtigungen beim Kauen und Beißen (ab 55 Jahre)*						
Nein	Ref.	–	Ref.	–	Ref.	–
Ja	4,2 (3,3–5,3)	< 0,001	3,8 (2,9–5,1)	< 0,001	4,0 (3,4–4,9)	< 0,001
*Zahnmedizinische Inanspruchnahme (12-MP)*						
Ja	Ref.	–	Ref.	–	Ref.	–
Nein	1,0 (0,7–1,3)	0,807	1,4 (1,1–1,9)	0,015	1,2 (0,9–1,4)	0,172
*Ungedeckter zahnmedizinischer Versorgungsbedarf (12-MP)*						
Nein	Ref.	–	Ref.	–	Ref.	–
Ja	2,2 (1,5–3,1)	< 0,001	2,7 (1,8–3,9)	< 0,001	2,3 (1,8–3,1)	< 0,001

*OR* Odds Ratios, *95 %-KI* 95 %-Konfidenzintervall, *Ref.* Referenzgruppe, *12-MP* 12-Monats-Prävalenz

## Diskussion

Die Ergebnisse aus GEDA 2019/2020-EHIS zeigen, dass etwas weniger als drei Viertel der Erwachsenen ihre Mundgesundheit als sehr gut oder gut einschätzten und etwas mehr als ein Viertel als mittelmäßig bis sehr schlecht. Internationale Studien liefern Prävalenzen, die zum Teil (deutlich) von den vorliegenden Ergebnissen abweichen (z. B. [6, 8, 12, 14, 19, 32]). Gründe hierfür sind Unterschiede in den Erhebungszeiträumen und einbezogenen Altersgruppen sowie in der Operationalisierung des Indikators hinsichtlich Fragestellung und Antwortkategorien. Darüber hinaus sind soziokulturelle Aspekte von Bedeutung, die die Mundgesundheit beeinflussen, wie z. B. unzureichende Fluoridexposition, ein erschwerter Zugang zu Mund- und Zahnpflegeprodukten oder ein eingeschränktes Angebot (zahn-)gesunder Lebensmittel [33]. Für eine Einordnung der Ergebnisse sind deshalb vor allem Studien aus Deutschland relevant.

Bundesweite und bevölkerungsrepräsentative Daten zur Mundgesundheit werden auch in der Deutschen Mundgesundheitsstudie (DMS) des Instituts der Deutschen Zahnärzte (IDZ) für ausgewählte Altersgruppen erfasst (35–44 Jahre, 65–74 Jahre, 75–100 Jahre) [34]. In der DMS wird die selbstwahrgenommene Mundgesundheit mit der Frage erhoben: „Wenn Sie an Ihre Zähne denken, wie ist der Zustand Ihrer Zähne?“ – „sehr gut“, „gut“, „zufriedenstellend“, „weniger gut“, „schlecht“ [34]. Laut einer Sonderauswertung des IDZ betrug der Anteil der Personen, die ihre Mundgesundheit als zufriedenstellend bis schlecht einschätzen, in der DMS V (2014) 54,4 % [35]. Ein direkter Vergleich mit den GEDA-Daten ist aufgrund von unterschiedlichen Erhebungszeiträumen und Altersgruppen nicht möglich. Zudem konzentriert sich die in den Studien gestellte Frage auf unterschiedliche Phänomenbereiche (DMS: Zähne, GEDA: Zähne und Zahnfleisch). Auch die vorgegebenen Antwortkategorien variieren in den beiden Studien deutlich. Dennoch ermöglichen die Ergebnisse der DMS vor dem Hintergrund der oben genannten soziokulturellen Unterschiede eine Orientierung an Daten aus Deutschland. Auch wenn die Ergebnisse nicht direkt vergleichbar sind, weisen beide Studien auf einen hohen Anteil von Personen hin, die ihre Mundgesundheit als nicht sehr gut oder gut einschätzen.

Männer beurteilten ihre Mundgesundheit häufiger als mittelmäßig bis sehr schlecht als Frauen. Dieser Geschlechterunterschied zeigte sich auch in internationalen Studien [7–9]. Als Erklärung können die Ergebnisse von Studien herangezogen werden, die zeigen, dass Männer im Vergleich zu Frauen häufiger eine unzureichende Mundhygiene aufweisen, seltener zahnärztliche Vorsorge- und Prophylaxeleistungen in Anspruch nehmen, sich seltener (zahn-)gesund ernähren und häufiger rauchen [30, 34, 36, 37].

Der Anteil der Personen mit einer mittelmäßigen bis sehr schlechten selbstwahrgenommenen Mundgesundheit nimmt bis ins späte mittlere Erwachsenenalter (45–64 Jahre) zu und verbleibt bis ins hohe Alter mit knapp über 30 % auf hohem Niveau. Während sich dieses Muster auch in einigen internationalen Studien zeigte [9, 10], stellten andere Studien fest, dass die Prävalenz einer schlechten selbstwahrgenommenen Mundgesundheit bis ins hohe Alter weiter zunimmt [7, 13, 14]. Dass die Mundgesundheit im Altersgang schlechter eingeschätzt wird, wird einerseits durch die Tatsache gestützt, dass Munderkrankungen wie Karies, Wurzelkaries und Parodontitis mit dem Alter häufiger auftreten – dies zeigt sich z. B. beim Vergleich der 35- bis 44-Jährigen und 65- bis 74-Jährigen in der DMS (für die Altersgruppe 45 bis 64 Jahre liegen keine Daten vor) [34]. Andererseits nimmt das Mundhygieneverhalten mit zunehmendem Alter ab und kontrollorientierte Zahnvorsorgeuntersuchen werden seltener in Anspruch genommen [9, 30, 34].

Beachtenswert sind auch die ausgeprägten Bildungsunterschiede, die in der selbstwahrgenommenen Mundgesundheit deutlich werden: Der Anteil der Personen mit einer mittelmäßigen bis sehr schlechten selbstwahrgenommenen Mundgesundheit verschlechterte sich zwischen den Bildungsgruppen um jeweils 5–10 Prozentpunkte. In der unteren Bildungsgruppe gab mehr als jede dritte Person eine mittelmäßige bis sehr schlechte Mundgesundheit an (36,6 %). Dieser Befund wird durch internationale Studien gestützt [7, 9–11, 14, 15]. Dieser Bildungsgradient spiegelt sich auch in der objektiven Mundgesundheit wider: Im Vergleich zu Personen höherer Bildungsgruppen sind Personen mit niedriger Bildung häufiger von Munderkrankungen wie Karies und Parodontitis betroffen [34]. Grund hierfür dürfte sein, dass Personen mit niedriger Bildung häufiger eine unzureichende Mundhygiene aufweisen, seltener kontrollorientierte Zahnvorsorgeuntersuchungen wahrnehmen, sich tendenziell seltener (zahn-)gesund ernähren und häufiger rauchen als Erwachsene höherer Bildungsgruppen [30, 34, 37].

Mit Blick auf den Zusammenhang zwischen der selbstwahrgenommenen Mundgesundheit und der Ernährung zeigen die Daten, dass der tägliche Verzehr zuckerhaltiger Getränke mit einer schlechteren selbstwahrgenommenen Mundgesundheit einherging. Dies ist nicht verwunderlich, da der Verzehr zuckerhaltiger Lebensmittel ein Risikofaktor für die Entstehung von Karies ist [38]. Ein nicht täglicher Konsum von Obst und Gemüse ist ebenfalls mit einer schlechteren selbstwahrgenommenen Mundgesundheit assoziiert. Dies zeigte sich bei getrennter Betrachtung sowohl für einen nicht täglichen Gemüsekonsum als auch einen nicht täglichen Obstkonsum (Daten dieser Sensitivitätsanalyse sind nicht gezeigt). Dies ist zunächst überraschend, da einige Obstsorten viel Fruchtzucker und Fruchtsäure enthalten, die den Zahnschmelz angreifen und so die Entstehung von Karies fördern [39]. Anzunehmen ist jedoch, dass ein täglicher Obstverzehr mit einer insgesamt gesünderen Ernährungs- und Lebensweise verbunden ist, wie z. B. einem geringeren Verzehr zuckerhaltiger Lebensmittel und dem Verzicht auf das Rauchen [40, 41]. Internationale Studien stützen die hier aufgezeigten Zusammenhänge zwischen der selbstwahrgenommenen Mundgesundheit und dem Ernährungsverhalten [13, 17].

Erwachsene, die täglich rauchen, schätzten ihre Mundgesundheit häufiger als mittelmäßig bis sehr schlecht ein als nicht rauchende Personen. Zu diesem Ergebnis kommen auch internationale Studien [14, 16, 18]. Rauchen schädigt durch die im Tabakrauch enthaltenen Schadstoffe auf vielfältige Weise die Mundhöhle: Bei Personen, die rauchen, treten Munderkrankungen wie Karies, Parodontitis und Krebs (Mundraum, Lippen, Zunge, Speicheldrüsen) häufiger auf als bei nicht rauchenden Personen [42]. Zudem kann Rauchen zu ästhetischen Veränderungen wie Verfärbungen an Zähnen, Lippen und Zunge führen. Laut den Ergebnissen ist das gelegentliche Rauchen nicht mit einer mittelmäßigen bis sehr schlechten Mundgesundheit assoziiert. Auch dieser Befund wird durch eine internationale Arbeit gestützt [16].

Für Erwachsene ab 55 Jahren wurde untersucht, ob Beeinträchtigungen beim Kauen und Beißen mit einer mittelmäßigen bis sehr schlechten selbstwahrgenommenen Mundgesundheit assoziiert sind. Ursache für Beeinträchtigungen beim Kauen und Beißen ist oftmals Zahnverlust – ein möglicher Endpunkt von Munderkrankungen wie Karies und Parodontitis im Erwachsenenalter [43, 44]. Von allen hier untersuchten Zusammenhängen ist dieser am stärksten ausgeprägt: Mehr als jede zweite Person, die beim Kauen und Beißen beeinträchtigt ist, gibt eine mittelmäßige bis sehr schlechte Mundgesundheit an (62,0 %). Das Risiko für eine mittelmäßige bis sehr schlechte selbstwahrgenommene Mundgesundheit ist bei diesen Personen um den Faktor 4,5 erhöht. Internationale Studienbefunde liegen auf ähnlichem Niveau [2, 11, 13].

Personen, die im Jahr vor der Befragung keine zahnärztliche oder kieferorthopädische Praxis aufgesucht haben, schätzten ihre Mundgesundheit häufiger als mittelmäßig bis sehr schlecht ein als Personen mit entsprechender Inanspruchnahme. Ein Zusammenhang zwischen einer schlechten selbstwahrgenommenen Mundgesundheit und dem letzten Zahnarztbesuch zeigte sich auch in internationalen Studien, die jeweils unterschiedliche Zeiträume für die Inanspruchnahme betrachtet haben (zwischen 1 und 3 Jahren vor der Befragung) [11, 12, 19, 20]. Je länger der letzte Zahnarztbesuch zurücklag, desto größer war das Risiko, die Mundgesundheit als schlecht einzuschätzen [20]. Andere Studien betrachteten den Zusammenhang zwischen selbstwahrgenommener Mundgesundheit und der Inanspruchnahme der kontrollorientierten Zahnvorsorgeuntersuchung. Die Ergebnisse zeigen erwartungsgemäß, dass die jährliche Inanspruchnahme einer derartigen Untersuchung das Risiko senkt, die Mundgesundheit als schlecht einzuschätzen [14, 15, 18].

Personen, die nach eigenen Angaben im letzten Jahr vor der Befragung eine zahnmedizinische Untersuchung oder Behandlung benötigt hätten, sich diese aber nicht leisten konnten, beurteilten ihre Mundgesundheit doppelt so häufig als mittelmäßig bis sehr schlecht wie Personen ohne solche Probleme. Dass ein (unerfüllter) zahnmedizinischer Versorgungsbedarf mit einer schlechteren selbstwahrgenommenen Mundgesundheit einhergeht, bestätigen auch internationale Studien [8, 10, 11]. Laut GEDA 2019/2020-EHIS bestand bei 9,6 % der Befragten den eigenen Angaben zufolge ein unerfüllter zahnmedizinischer Versorgungsbedarf (eigene Berechnung). Auch andere Studien haben den unerfüllten zahnmedizinischen Versorgungsbedarf in Deutschland untersucht [31, 45, 46]. Dabei zeigten sich große soziale Unterschiede. Finanzielle Gründe waren die häufigste Ursache, warum notwendige zahnmedizinische Behandlungen nicht wahrgenommen wurden.

Laut den Ergebnissen des multivariaten Gesamtmodells waren Beeinträchtigungen beim Kauen und Beißen erwartungsgemäß am stärksten mit einer mittelmäßigen bis sehr schlechten selbstwahrgenommenen Mundgesundheit assoziiert (OR 4,0). Dies liefert Hinweise darauf, dass Menschen ihre Mundgesundheit relativ gut einschätzen können. Die selbstwahrgenommene Mundgesundheit wäre somit ein geeigneter Indikator, um im Rahmen von Befragungssurveys mit einem im Vergleich zu Untersuchungssurveys geringeren Erhebungsaufwand Informationen zum Mundgesundheitszustand der Bevölkerung zu erhalten. Darüber hinaus war im multivariaten Gesamtmodell ein unerfüllter zahnmedizinischer Versorgungsbedarf relativ stark mit einer mittelmäßigen bis sehr schlechten selbstwahrgenommenen Mundgesundheit assoziiert (OR 2,3). Dieses Ergebnis spricht für wahrgenommene Barrieren im Gesundheitssystem. Nach statistisch wechselseitiger Kontrolle aller Merkmale zeigten sich zudem moderate Zusammenhänge zwischen einer mittelmäßigen bis sehr schlechten selbstwahrgenommenen Mundgesundheit und männlichem Geschlecht (OR 1,5), einem täglichen Obst- und Gemüsekonsum (OR 1,2) sowie bei Männern für eine niedrige Bildung (OR 2,1), tägliches Rauchen (OR 1,6) und eine nicht jährliche zahnmedizinische Inanspruchnahme (OR 1,4). Trotz moderater Zusammenhänge geben diese Ergebnisse Hinweise auf das präventive Potenzial dieser Determinanten für die Mundgesundheit.

### Limitationen

Die Angaben zur selbstwahrgenommenen Mundgesundheit erlauben eine allgemeine Einschätzung, aber keine differenzierte Beurteilung, weil die angewendete Frage nur einen Teilaspekt der Mundgesundheit erfasst. Weitere Aspekte, wie z. B. die Mundschleimhaut, sind in der Fragestellung nicht enthalten. Die Recherche von assoziierten Faktoren der selbstwahrgenommenen Mundgesundheit hatte ergeben, dass eine unzureichende Mundhygiene ein wichtiger Risikofaktor ist. Dieser Zusammenhang kann mit den vorliegenden Daten nicht untersucht werden, da in GEDA 2019/2020-EHIS keine entsprechenden Informationen erhoben wurden. Diese Arbeit konnte zeigen, dass eine mittelmäßige bis sehr schlechte selbstwahrgenommene Mundgesundheit mit der Selbstangabe eines unerfüllten zahnmedizinischen Versorgungsbedarfs einhergeht. Ob dieser Bedarf tatsächlich vorlag, kann nicht überprüft werden. Darüber hinaus ist unklar, welche Art der zahnmedizinischen Behandlung sich die Befragten nicht leisten konnten. Dies wäre eine wichtige Information, da die Höhe der Kosten für diese Behandlungen durchaus variieren kann [47]. Abschließend ist anzumerken, dass bestimmte chronische Erkrankungen, wie z. B. Diabetes mellitus, die Mundgesundheit negativ beeinflussen können. Zukünftige Analysen zur selbstwahrgenommenen Mundgesundheit sollten daher auch krankheitsbezogene Aspekte berücksichtigen.

### Ansatzpunkte zur Förderung der Mundgesundheit

Mit den vorliegenden Auswertungen konnten Faktoren identifiziert werden, die mit einer mittelmäßigen bis sehr schlechten selbstwahrgenommenen Mundgesundheit assoziiert sind. Diese weisen auf mögliche Ansatzpunkte bei der Verbesserung und dem Erhalt der Mundgesundheit hin. Hierzu zählen neben einer angemessenen Mundhygiene und regelmäßigen kontrollorientierten Zahnvorsorgeuntersuchungen, eine (zahn-)gesunde Ernährung und das Nichtrauchen. Dies steht im Einklang mit dem bisherigen Wissensstand [1]. Die vorliegenden Analysen konnten darüber hinaus Gruppen identifizieren, deren Risiko für eine mittelmäßige bis sehr schlechte selbstwahrgenommene Mundgesundheit erhöht ist. Dazu zählen Männer, Personen ab dem späten mittleren Erwachsenenalter und Personen mit niedriger Bildung.

Bei der Vermittlung von Empfehlungen zur Veränderung des Gesundheitsverhaltens zur Verbesserung bzw. zum Erhalt der Mundgesundheit spielen Zahnärztinnen und Zahnärzte eine wichtige Rolle. Das gilt sowohl für die verhaltensbezogenen Aspekte als auch für die Aufklärung über bestehende Ansprüche und möglicherweise anfallende Kosten [48]. Studien konnten zeigen, dass eine schlechte selbstwahrgenommene Mundgesundheit mit einer geringen Mundgesundheitskompetenz assoziiert ist [8, 49, 50], also der Fähigkeit, mundgesundheitsrelevante Informationen zu verstehen, zu verarbeiten und anzuwenden. Somit ist wichtig, die Mundgesundheitskompetenz zu stärken und das Wissen über die Bedeutung eines angemessenen Mundgesundheitsverhaltens zu verbessern. Eine Strategie zur Förderung der Mundgesundheitskompetenz wurde 2017 von der Kassenzahnärztlichen Bundesvereinigung (KZBV) vorgelegt [48] und die Bundeszahnärztekammer (BZÄK) unterstützt seit 2017 die „Allianz für Gesundheitskompetenz“ auf Bundesebene [51]. Zentrales Ziel ist, eine wirksame Kommunikation und Informationsvermittlung in der Zahnarztpraxis zu schaffen, auch mit Blick auf das Mundgesundheitsverhalten, wie z. B. bei der Mundhygiene und der Ernährung [48, 51]. Dabei sollten weitere Aspekte, wie z. B. das Thema Rauchen, berücksichtigt werden. Auch die umfangreichen Regelungen zu Eigenanteilen und Festzuschüssen bei zahnmedizinischen Behandlungen sollten verständlich und umfassend erläutert werden, auch um einem ggf. unerfüllten Bedarf an zahnmedizinischer Versorgung zu begegnen. Wichtig ist, dass Eigenanteile zu zahnmedizinischen Behandlungen finanzierbar sind, um sozial bedingte Ungleichheiten in der Mundgesundheit zu verringern [45].

Der Befund, dass Personen ohne zahnmedizinische Inanspruchnahme innerhalb des letzten Jahres häufiger eine mittelmäßige bis sehr schlechte Mundgesundheit berichten, führt zu der Frage, wie diese Personen besser erreicht werden können. Zahnärztlich-hausärztliche Kooperationen könnten hierzu einen Beitrag leisten, indem Hausärztinnen und Hausärzte bspw. ihre Patientinnen und Patienten zu regelmäßigen Besuchen in der Zahnarztpraxis motivieren [30].
